# Hypertrophy of the Clitoris

**Published:** 1895-10

**Authors:** A. W. Clement

**Affiliations:** Baltimore, Md.


### HYPERTROPHY OF THE CLITORIS.

By A. W. CLEMENT, V.S.,
BALTIMORE, MD.

Subject, a full-bred hackney filly, two years old, well developed; was observed to be apparently in heat during the latter part of last spring. Upon special examination the lips of the vulva were seen to be constantly open, and what appeared to be the clitoris, enlarged to double its normal size, constantly protruded. Hot water was applied, and the vagina well syringed out daily with a mild solution of subacetate of lead. This had the effect of somewhat, though very slightly, reducing the swelling. Belladonna ointment was afterward applied, but without effect. She was then served by a hackney stallion, though the service was not very close on account of the partial obstruction, and the filly bled quite freely after the service. She has refused the horse ever since, but the enlargement remained the same. Not anticipating anything out of the ordinary, she was turned loose with a lot of brood mares. She continually tried to mount them, and when successful would go through the motion of service. The enlargement would become erect, would protrude from the vulva for four or five inches, and at the culmination of the act she would eject a quantity of fluid. She could tease a mare as well as any stallion, and would become greatly excited at the sight of a mare. All of this time she refused the stallion after the first service. Diagnosis of hypertrophied clitoris made and amputation advised. The operation was performed about the first of August. Since that time she has not shown any amorous disposition, and the wound is quite healed. It will be interesting to note whether there is any return of the perverted sexual desire, and whether or not she proves in foal.

